# Supplementary material for: Comparison of Structured Nutrition Therapy for Ramadan with Standard Care in Type 2 Diabetes Patients
Source: Nutrients. 2020 Mar 19;12(3):813. doi: 10.3390/nu12030813 (PMC7146585; doi:10.3390/nu12030813)
Supplement: Supplementary file 1 [file nutrients-12-00813-s001.pdf]

**Table S1.** Energy intake recommendation and carbohydrate exchange distribution during Ramadan

| **Energy intake (kcal/day)** | | **1200** | **1500** | **1800** |
|---|---|---|---|---|
| **Recommended group** | | Women with height <150 cm (weight loss) | Women with height <150 cm (weight maintenance), or women with height >150cm (weight loss/ maintenance) | Men (weight loss/ maintenance) |
| **Carbohydrate exchanges** | | **10** | **12** | **14** |
| **Distribution between meal times** | ***Suhoor*** | 3 | 4 | 5 |
| | **Snack 1** | 2 | 2 | 2 |
| | ***Iftar*** | 3 | 4 | 5 |
| | **Snack 2** | 2 | 2 | 2 |

Table modified from IDF and DAR International Alliance Guidelines [2]
Snack 1: a small snack before *Iftar* (also called "Mini *Iftar*")
Snack 2: pre-bed snack, typically taken after *Taraweeh* prayer

**Table S2.** Daily energy intake distribution during Ramadan

| **Mealtime** | **%TEI** |
|---|---|
| ***Suhoor*** | 30-40 |
| **Snack 1** | 10-20 |
| ***Iftar*** | 30-40 |
| **Snack 2** | 10-20 |

%TEI, percentage from total energy intake

**Table S3.** Sample menu for one day

| **Energy intake distribution** | **1200 kcal/day** | **1500 kcal/day** | **1800 kcal/day** |
|---|---|---|---|
| *Suhoor* **(30-40%TEI)** | **Fried rice with egg**<br>• Brown rice, 1 cup<br>• Egg, 1 whole<br>• Carrot and long beans, 1 cup<br>**Vegetable salad**<br>• Lettuce, tomato and cucumber – 1 ½ cup<br>**DSF**, 3 ½ scoops + 120 ml water<br>(~400 kcal, 3 CHO exchanges) | **Fried rice with egg**<br>• Brown rice, 1 cup<br>• Egg, 1 whole<br>• Carrot and long beans, 1 cup<br>**Vegetable salad**<br>• Lettuce, tomato and cucumber – 1 ½ cup<br>**DSF**, 7 scoops + 210 ml water<br>(~530 kcal, 4 CHO exchanges) | **Fried rice with egg**<br>• Brown rice, 1 ½ cup<br>• Egg, 1 whole<br>• Chicken breast, 1 piece (40g)<br>• Carrot and long beans, 1 cup<br>**Vegetable salad**<br>• Lettuce, tomato and cucumber – 1 ½ cup<br>**DSF**, 7 scoops + 210 ml water<br>(~685 kcal, 5 CHO exchanges) |
| **Snack 1 (10-20%TEI)** | **Dragon fruit DSF shake**<br>• Dragon fruit, 3 slices (94g)<br>• DSF, 3 ½ scoops + 120 ml water<br>(~195kcal, 2 CHO exchanges) | **Dragon fruit DSF shake**<br>• Dragon fruit, 3 slices (94g)<br>• DSF, 3 ½ scoops + 120 ml water<br>(~195kcal, 2 CHO exchanges) | **Dragon fruit DSF shake**<br>• Dragon fruit, 3 slices (94g)<br>• DSF, 3 ½ scoops + 120 ml water<br>(~195kcal, 2 CHO exchanges) |
| *Iftar* **(30-40%TEI)** | **Rice with side dishes**<br>• Brown rice, 1 cup | **Rice with side dishes**<br>• Brown rice, 1 ½ cup | **Rice with side dishes**<br>• Brown rice, 2 cups |

| | | | |
|---|---|---|---|
| | • Spanish mackerel curry, 1 piece (66g raw)<br>• Pak choi, cooked, 1 cup<br>• Stir fried tofu (1 piece, 64g) and beansprouts (1/2 cup)<br>**Kiwi,** 1 whole (100g)<br>**Plain water**<br>(~375kcal, 3 CHO exchanges) | • Spanish mackerel curry, 1 ½ piece (99g raw)<br>• Pak choi, cooked, 1 cup<br>• Stir fried tofu (1 piece, 64g) and beansprouts (1/2 cup)<br>**Kiwi,** 1 whole (100g)<br>**Plain water**<br>(~530kcal, 4 CHO exchanges) | • Spanish mackerel curry, 1 ½ piece (99g raw)<br>• Pak choi, cooked, 1 cup<br>• Stir fried tofu (1 piece, 64g) and beansprouts (1/2 cup)<br>**Kiwi,** 1 whole (100g)<br>**Plain water**<br>(~710kcal, 5 CHO exchanges) |
| **Snack 2 (10-20%TEI)** | ***Roti jala***<br>• *Roti jala,* 2 rolls<br>• Dhal curry, ½ cup (90g)<br>**Plain water or tea/coffee without sugar**, 1 cup<br>(~215kcal, 2 CHO exchanges) | ***Roti jala***<br>• *Roti jala,* 2 rolls<br>• Dhal curry, ½ cup (90g)<br>**Plain water or tea/coffee without sugar**, 1 cup<br>(~215kcal, 2 CHO exchanges) | ***Roti jala***<br>• *Roti jala,* 2 rolls<br>• Dhal curry, ½ cup (90g)<br>**Plain water or tea/coffee without sugar**, 1 cup<br>(~215kcal, 2 CHO exchanges) |
| **Total CHO exchanges** | 10 | 12 | 14 |

%TEI, percentage from total energy intake; CHO, carbohydrate; kcal, calories; DSF, diabetes specific formula
*Roti jala*: A popular Malaysian snack typically served with curry; similar texture with soft crepe
DSF: 3 ½ scoops constitute 1 exchange of carbohydrate

**Table S4.** Ramadan nutrition plate composition

| Plate composition | Details |
|---|---|
| ¼ Carbohydrates | • Includes rice, noodle, bread, grain, grain products<br>• Choose whole-grain, high fiber, and low in GI carbohydrates |
| ¼ Protein | • Includes fish, chicken, beef, and plant protein<br>• Choose lean beef/poultry cuts<br>• Choose low fat cooking method |
| ½ Vegetables | • Includes non-starchy vegetables such as carrot, tomatoes<br>• Eat vegetables before carbohydrates |
| Soup or gravy | • Choose low fat cooking method |
| Fruit | • Choose fresh fruit or dates |
| Water | • Choose plain water<br>• Reduce intake of caffeinated drink |
